# Supplementary figures and images for: Transcriptome Response of Differentiating Muscle Satellite Cells to Thermal Challenge in Commercial Turkey
Source: Genes (Basel). 2022 Oct 14;13(10):1857. doi: 10.3390/genes13101857 (PMC9601516; doi:10.3390/genes13101857)

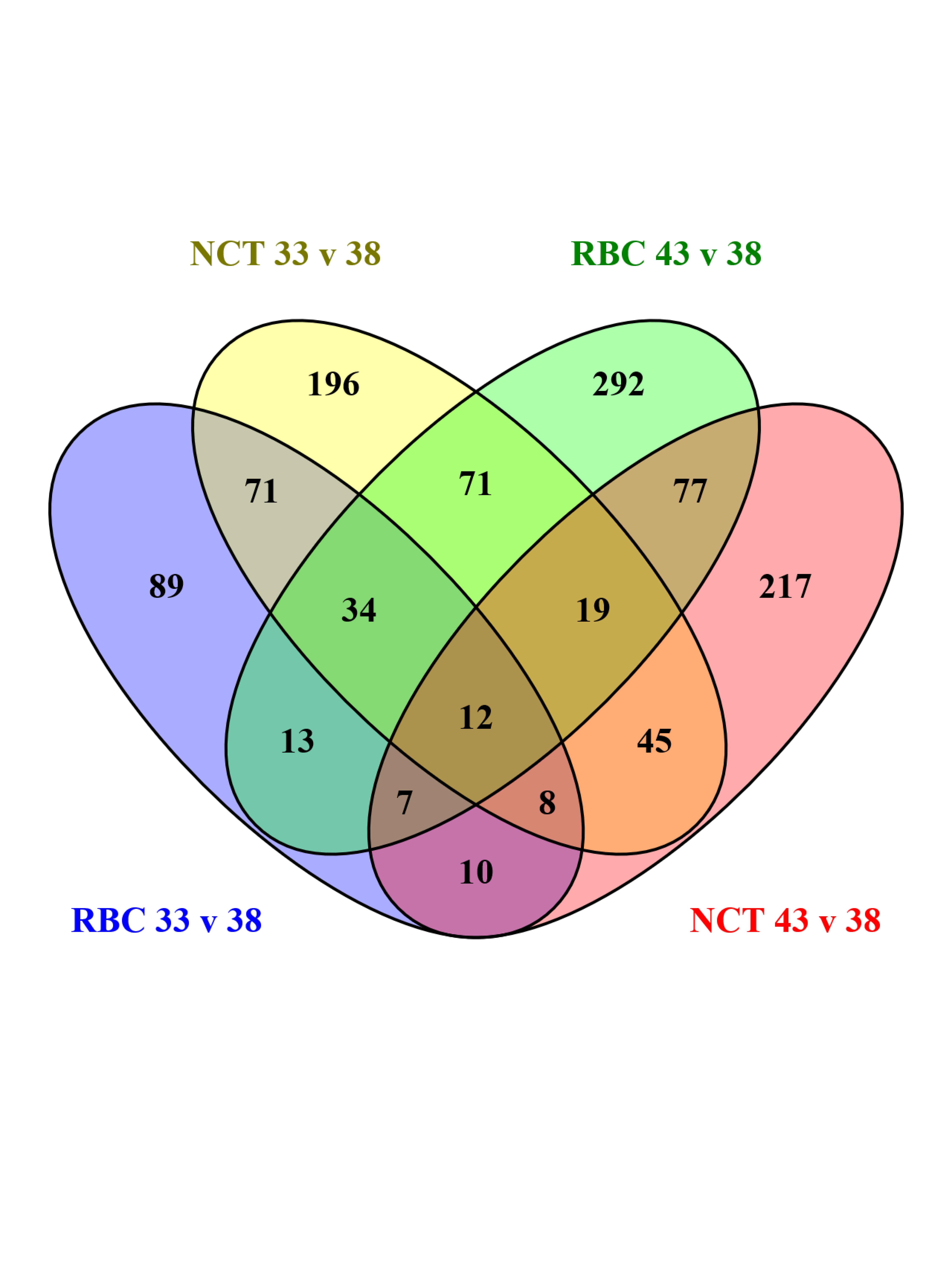

Supplement: Supplementary file 1 [file genes-13-01857-s001.zip › Figure S1.tiff]
